# The Nordic maintenance care program: the clinical use of identified indications for preventive care

**DOI:** 10.1186/2045-709X-21-10

**Published:** 2013-03-06

**Authors:** Iben Axén, Lennart Bodin

**Affiliations:** 1 Intervention & Implementation Research, Institute of Environmental Medicine, Karolinska Institutet, Nobels väg 13, Stockholm, 171 77, Sweden

## Abstract

**Background:**

Low back pain (LBP) is a prevalent condition and has been found to be recurrent and persistent in a majority of cases. Chiropractors have a preventive strategy, maintenance care (MC), aimed towards minimizing recurrence and progression of such conditions. The indications for recommending MC have been identified in the Nordic countries from hypothetical cases. This study aims to investigate whether these indications are indeed used in the clinical encounter.

**Methods:**

Data were collected in a multi-center observational study in which patients consulted a chiropractor for their non-specific LBP. Patient baseline information was a) previous duration of the LBP, b) the presence of previous episodes of LBP and c) early improvement with treatment. The chiropractors were asked if they deemed each individual patient an MC candidate. Logistic regression analyses (uni– and multi-level) were used to investigate the association of the patient variables with the chiropractor’s decision.

**Results:**

The results showed that “previous episodes” with LBP was the strongest predictor for recommending MC, and that the presence of all predictors strengthens the frequency of this recommendation. However, there was considerable heterogeneity among the participating chiropractors concerning the recommendation of MC.

**Conclusions:**

The study largely confirms the clinical use of the previously identified indications for recommending MC for recurrent and persistent LBP. Previous episodes of LBP was the strongest indicator.

## Background

In the past few decades, the prevalence of low back pain, LBP, has been found to be extremely high [1] and the resulting costs of the condition are substantial [2]. Upon further scrutiny, the condition has been found to be recurrent in most cases and persistent in some [3-5]. These facts invite preventive approaches, both from a personal and societal perspective. Secondary prevention, to minimize the recurrences or the impact of episodic LBP, and tertiary prevention, to minimize the effects of persistent LBP, seem warranted.

In the chiropractic profession, there is a traditional preventive approach named Maintenance Care, MC. It has been defined as: “…treatment, either scheduled or elective, which occurred after optimum recorded benefit was reached” [6] and “a regimen designed to provide for the patient’s continued well-being or for maintaining the optimum state of health while minimizing recurrences of the clinical status” [7]. However, a review concluded that there is no evidence-based definition, no identified indications for use nor evidence of effect of MC [8]. During the past decade, efforts have been made in the Nordic countries to describe the intent [9,10], content [9,10] and frequency [10,11] of this approach. In the US, efforts have been made to develop consensus definitions regarding this practice [12].

The indications for MC have also been studied in a series of studies through a process of triangulation. In short, the indications were identified in qualitative focus group discussions [13], and then tested in questionnaires across the Nordic countries [11,13,14]. As a third step, case management strategies were explored to investigate chiropractors’ decisions using hypothetical but clinically relevant cases in a questionnaire [15] as well as in an interview study [16]. During the process, clinicians argued that it was difficult for them to identify the most important indicator, as several factors will always be considered in the clinical encounter. However, when asked to grade the suggested factors, the chiropractors in Sweden, Finland and Denmark agreed that secondary prevention would be recommended to a patient who reported previous episodes of the condition, and that the indication for tertiary care was improvement with treatment [13]. Further, the practice of using preventive strategies seemed similar in the Nordic countries, albeit there seemed to be a group of clinicians who seemed to use MC to a larger extent than most [15].

As these indications have been identified through hypothetical cases they are, in that sense, theoretical constructs. Whether they represent clinical reality is still unknown. This study aimed to test if these theoretically defined indications are really in use in a clinical setting/situation. To test the efficacy of MC in future studies in the clinical setting, it is important to know what indications are actually used. This will ensure that the relevant subgroups of patients are included, i.e. the subgroups that chiropractors usually recommend MC to. It will then be possible to study if the outcome of the MC treatment is associated with these criteria.

## Methods

The data stems from a multicenter observational study in which 262 patients consulting for LBP were followed for six months. Thirty-three chiropractors were involved in the data collection, which took place between May 2007 and September 2008. The study procedures are described elsewhere [17]. In this study, only data collected at baseline and at the 4th visit were used.

At baseline, variables concerning previous duration and previous episodes were collected. At the 4th consultation, information regarding self-rated improvement (5 graded scale: Definitely worse, probably worse, unchanged, probably better and definitely better) and the chiropractors’ opinion regarding MC (was this a patient to whom MC would be recommended?) were collected. The baseline and 4th visit variables were dichotomised as follows: long or short previous duration (more than and less than 30 pain days, respectively), few or many previous episodes (less than 4 and 4 episodes or more the previous 2 years, respectively), improved and not improved (definitely better vs. all the other categories) and MC candidate (yes or no).

Predictive models were analyzed in which the independent variables duration, episodes and improvement (alone or in different combinations) were tested against the dependent variable “MC candidate”. The hypothesis was that a patient with a) long previous duration, b) many previous episodes and c) definite improvement at the 4th visit would be an MC candidate. Consequently, a patient with short previous duration, few previous episodes and not reporting improvement by the 4th visit would not be an MC candidate. Our analyses aimed to apply the independent variables in univariate models as well as in different combinations in multivariate models. Thus we hypothesized that a dose–response relationship would be present (a combination of two predictors would lead to a MC recommendation more often than only one). The participating clinicians were blind to the study hypothesis as they were told that we wanted to observe what really goes on in clinical practice. To investigate the “chiropractor effect” on the MC decision, we applied additional analytic models where a random variable for chiropractors was added, thus incorporating the hypothesized random variation between chiropractors in their recommendations for MC.

The data were initially described in a cross-table showing the presence of MC against the predictor variables and then analyzed using two different logistic regression models. In the first specification of these models only the patient dimension of the data was used. Analyses were done with each predictor variable separately and then in a multiple analysis with all three variables included. We refer to this analysis as an ordinary logistic regression model or one-level model. In our second specification the chiropractor variable was used as a random variable and the data thus followed a two-level structure, the patient level with 252 subjects and the clinician level with 33 chiropractors. We refer to this analysis as a multilevel model with two levels. For both specifications of the logistic models we also analyzed if interactions between the predictor variables were of statistical significance. In addition, the models were applied with stratification for gender. The outcome parameter was the odds ratio (OR), shown with 95% confidence interval (CI). Model fit was summarized in Akaike’s index [18] where smaller values show a better fit to the data. The AIC index may be used to compare competing models [19], as the criterion was developed to find an optimal and at the same time parsimonious model, not entering unnecessary variables [20]. It is stated that a difference in AIC of more than two units indicates a marked preference for the model with a smaller criterion measure [19].

As an additional measure of model fit and ability to predict an outcome the ROC curves [21] were drawn. Statistical significance was regarded as present if p < 0.05. All analyses were performed using SPSS v 20 [22] and STATA v 12 [23].

The study was approved by the local ethics committee at the Karolinska Institutet: 2007/1458-31/4. All the participating clinicians and patients signed informed consent forms.

## Results

The sample is extensively described elsewhere [17]. Complete data on 252 subjects were available. In short, the participants were on average 44 years old with a fairly even gender distribution (52% male). The LBP was rated on a numeric rating scale, NRS [24] at 4.4/10 at baseline. Table 1 lists the major features of the sample.

**Table 1:** Description of the study sample (N = 252)

**Variable/outcome**	**Frequency/mean**
Gender, male	52%
Age, mean	44 (SD 11.6)
Pain intensity, NRS Mean	4.4 (SD 2.2)
Duration ≥30 days previous year	57%
Episodes ≥4 previous 2 years	47%
Definitely better by the fourth visit	71%
MC indicated	80%

The cross tabulation of predictors and the MC recommendation revealed that patients with three predictors (long previous duration, many previous episodes and definite improvement by the fourth visit) were regarded as MC candidates in 93.4% of cases. This group amounted to 24% of the total sample. On the other end of the scale, patients with no predictors were regarded as MC candidates in 73% of the cases. This group, however, amounts to only 6% of the total sample.In cases where two predictors were present, patients were regarded as MC candidates more often than when only one predictor was present. The different combinations of predictors and the MC decisions are listed in Table 2.

**Table 2:** Cross tabulations of predictive indicators and the outcome “Maintenance Care (MC) recommendation”

**Predictors**	**MC –yes (N)**	**Total % (N)**	**Number of predictors**	**MC-yes (N)**
**Long duration +**				
**Many episodes +**	93.4% (57)	24 (61)	3	93.4% (57)
**Definitely better**				
**Long duration +**				
**Many episodes +**	86.8% (33)	15 (38)	2	83.3% (70)
NOT better				
SHORT duration +				
**Many episodes +**	84.6% (11)	5 (13)		
**Definitely better**				
**Long duration +**				
FEW episodes +	78.8% (26)	13 (33)		
**Definitely better**				
SHORT duration +			1	68.8% (64)
**Many episodes +**	87.5% (7)	3 (8 )		
NOT better				
**Long duration +**				
FEW episodes +	61.5% (8)	5 (13)		
NOT better				
SHORT duration +				
FEW episodes +	68.1% (49)	28 (72)		
**Definitely better**				
SHORT duration +				
FEW episodes +	73.3% (11)	6 (15)	0	73.3% (11)
NOT better				

Outcome of the predictors in bold are à priori expected to be associated with recommendations for MC.

Estimates from the two regression models are shown in Table 3. The predictor “many previous episodes” shows statistical significance in both the regression models. It is evident that the multilevel specification has a considerably better fit to data, that is, the chiropractors show a non-ignorable heterogeneity in their recommendation of MC. Adding interaction factors for the predictive variables did not reach statistical significance in any of the models.

**Table 3:** Logistic regression models for prediction of the Maintenance Care (MC) -recommendation

	**Ordinary (one level**^**1**^**) Logistic regression**			**Multi-level (two levels**^**1**^**) Logistic regression**		
**Factors**	**OR**	**95% CI**	**p-value**	**OR**	**95% CI**	**p-value**
Many previous episodes	3.5	1.6 – 7.7	0.002	4.1	1.5 – 10.7	0.005
Long duration	1.4	0.7 – 2.8	0.39	1.5	0.6 – 3.6	0.35
Definitely better by the 4th visit	1.4	0.7 – 3.0	0.33	1.2	0.5 – 3.0	0.62
Akaike’s Index (AIC)^2^	244.69				225.41						

^1^ The levels are patients (n = 252) for the one-level model and patients (n = 252) and chiropractors (n = 33) for the two-level model. ^2^ A smaller value for AIC indicates better fit to data.

**Figure 1:**
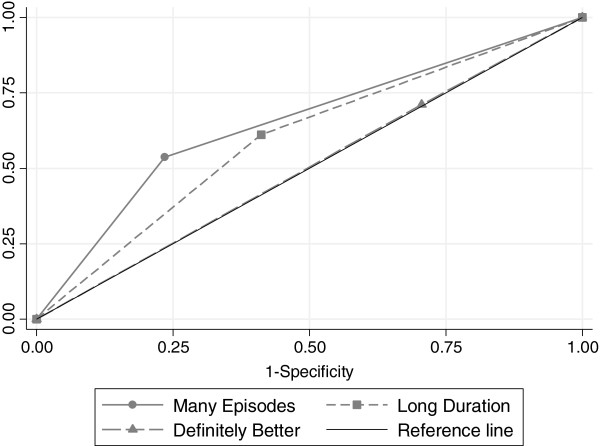
**Three ROC curves for the investigated predictors for the MC-recommendation together with a reference line for no discrimination (a random predictive capability).** Areas under the ROC curves are 0.65 for Many Episodes, 0.60 for Long Duration and 0.50 for Definitely Better, the latter almost coinciding with the reference line.

Our subsequent analyses are summarized in Table 4 where the model fit in multilevel models is described by Akaike’s index. In the table the three univariate regressions for each one of the predictor variables are shown first, followed by a model where the independent variable is the sum of ‘positive’ predictor variables. Finally the multiple regression model with all three predictive variables simultaneously included is shown. The best fit is by this index reported by one of the simplest models, where only the variable “many previous episodes” is included (AIC = 222.5). Properties of the three predictor variables with respect to their sensitivity and specificity in relation to the MC recommendation are shown in Figure 1. The variable “many previous episodes” has the highest value for the area under the ROC curve, although it has a somewhat lower sensitivity than the other two variables, but it is considerably better with respect to specificity. The stratification according to gender of the patients did not reveal any significant differences (results not shown).

**Table 4:** Model fit for multi-level logistic regressions for the separate predictive factors, for the number of predictive factors and for all three predictive indicators simultaneously analysed

**Predictive factor**	**Model fit by Akaike’s index AIC**^**1**^
Many previous episodes	222.5
Long duration	230.1
Definitely better by the 4th visit	236.6
Number of predictive factors (0–3)	226.6
Long duration, Many previous episodes, Definitely better by the 4th visit.	225.4

^1^ A smaller value for AIC indicates better fit to data.

## Discussion

In this study, the decisions of chiropractic clinicians to recommend secondary and tertiary preventive care, MC, for recurrent and persistent LBP were tested using theoretically defined indications. We tested models in which three, two, one and none of the indications were used. We propose that, in clinical reality, this information is weighed together consciously or subconsciously to form a clinical decision.

The results largely confirm the findings of the previous studies in the area [10,13,14]. That is, the theoretical construct previously identified was found to reflect reality. The clinical encounter is always tailored to the individual patient, but clinicians are clearly using some overarching principles when recommending MC. The clinicians in this study weigh these factors together when deciding on MC. The presence of many previous episodes was found to be the main indicator for such care in the clinical encounter. This might suggest that clinicians are viewing MC mainly as secondary prevention aimed at preventing future episodes, and is in line with the MC intent described in previous studies [10,13,14].

The accuracy of the predictive models was examined using ROC curves. The area under the ROC curve for the factor “many previous episodes”, suggests that the predictive accuracy of this model is better than “long duration” and far better than “definite improvement”. It is interesting that the sensitivity of the best model is less accurate than the specificity. Thus, the absence of many previous episodes more accurately predicts a decision not to recommend MC (specificity 0.72), than the presence of many previous episodes predicts a MC recommendation (sensitivity 0.53). From previous studies, it is known that clinicians consider a number of factors before recommending MC, factors such as psychosocial situation, work demands, patient motivation and so on [13]. We did not record these variables in this study, nor can we know if clinicians use some other, maybe tacit, knowledge in their decision process.

By adding the clinicians as a factor in the multi-level regression model, the model fit was improved. We conclude that the heterogeneity among chiropractors in regards to recommending MC is substantial. This is also in line with the findings of a previous study [15].

Further, the initial hypothesis was not confirmed in full, as not all patients with three predictors were regarded as MC candidates and a majority of patients with no predictors also were given this recommendation (keeping in mind that the latter is a small group). Again, it is possible that some other unknown or unrecorded variables were considered in these cases, making the decisions go either way depending on the type and presence or absence of that information. This could possibly explain the fact that even the patients with no predictors to a large extent (73%) were regarded as MC candidates. A previous study explained the clinician’s intent of continuing treatment despite the lack of progress in terms of taking on the role as a health coach [25]. Further, a recent consensus process among the chiropractic profession described “wellness care” with a primary preventive intent: to promote general health including counseling on behavior related to diet, exercise and tobacco [12]. We did not investigate these aspects of the MC decision, and this subgroup (with no predictors) was very small, rendering conclusions subject to caution.

It is important to note that we do not know if the patients involved in this study that were deemed “MC candidates” were actually given the MC recommendation, if they accepted it and what the outcome of that preventive strategy was.

The major limitation of this study is the scarcity of variables, which is a result of time restrictions in the clinic. The objective was to test a theoretical construct, which was possible using the available data which are part of the normal clinical encounter. However, it would have been possible to add an explorative element to the study with data concerning psychosocial factors, motivation, work demands etc. which would possibly add an explanatory value to the results. Still, as the main hypothesis was largely confirmed, the theoretical construct was found to be reflective of reality to some extent. The results are also restricted by the detail of the available data. Had more categories been added to previous duration and previous episodes, detailed associations regarding subgroups may have been explored. Both variables are self-reported and may be subject to memory bias. For previous duration, the cut point of 30 days the previous year has been used in several studies [26-28] and found to be useful in separating patients with good and poor long term prognosis. For episodes, no evidence-based definition exists [29], and the decision to ask the patients to remember whether they had many (≥4) or fewer (<4) was based upon discussions with clinicians.

## Conclusions

The previously identified indications for recommending MC are indeed being used in the clinicians’ decision-making. When the patient is presenting with a history of back pain (>30 days the previous year), many (≥4) previous episodes and definite improvement by the fourth chiropractic visit, the overwhelming majority of clinicians (93%) would consider recommending MC. The strongest indicator for this recommendation was the presence of many previous episodes. However, the model also indicated heterogeneity among clinicians in making this decision.

The results of this study may be used in future studies designed to test the efficacy of MC, in order to include the clinically relevant subgroup of patients.

## Competing interests

The authors declare that they have no competing interests.

## Authors’ contributions

IA was responsible for the study from which the data were extracted. She was responsible for the interpretation of the results and for writing the first manuscript draft. LB was responsible for the statistical analyses and their interpretation. Both authors read and approved the final manuscript.
